# Supplementary material for: Cellular production of a de novo membrane cytochrome
Source: Proc Natl Acad Sci U S A. 2023 Apr 10;120(16):e2300137120. doi: 10.1073/pnas.2300137120 (PMC10120048; doi:10.1073/pnas.2300137120)
Supplement: Supplementary file 1 — Appendix 01 (PDF) [file pnas.2300137120.sapp.pdf]

Supplementary Information for:

Cellular production of a *de novo* membrane cytochrome

Benjamin J Hardy[1,2], Alvaro Martin Hermosilla[1], Dinesh K Chinthapalli[3], Carol V Robinson[3], JL Ross Anderson[1,2], Paul Curnow[1,2‡]

[1]School of Biochemistry, University of Bristol, UK; [2]BrisSynBio, Life Sciences Building, Tyndall Avenue, Bristol, UK; [3]Department of Chemistry, University of Oxford, UK

[‡]Corresponding author: p.curnow@bristol.ac.uk

<u>**Contents**</u>

Supplementary methods
Molecular dynamics simulations

Figure S1: Sequence logo presentation of the multiple sequence alignment of the 200 best-scoring decoys from Rosetta design.
Figure S2: Comparison of surface-swapped amino acid sequences produced by Rosetta design.
Figure S3: Synthetic genes corresponding to each protein design.
Figure S4. Purification of three Rosetta designs.
Figure S5. Topology prediction and *ab initio* structure prediction of CytbX.
Figure S6. Molecular dynamics simulations of CytbX in an explicit bilayer.

Supplementary Methods

Molecular Dynamics simulations

Relaxed CytbX model structures were inserted into 3:1 DOPE:DOPG lipid bilayers using PACKMOL-Memgen[1] with bis-histidine coordination of *b*-type hemes as described[2]. The systems were protonated using Reduce, and solvated and parameterised using LEaP. The ff14SB forcefield was used for protein, Lipid17 for lipids, and tip3p for water and ions[3,4]. All molecular dynamics simulations were run using the Amber18 software package[5,6] with GPU acceleration[7]. Systems were energy minimized by three rounds of 5000 steps steepest-descent minimization, with harmonic restraints for protein non-hydrogen atoms only, for protein C-alpha atoms only and with no restraints, respectively. Systems were then heated to 303K over 100 ps using Langevin dynamics and 1 Bar pressure was then applied using a Berendsen barostat[8] with semi-isotropic pressure coupling over a further 100 ps (2 ps pressure relaxation). Using GPU acceleration, systems were further equilibrated for 100 ps at 1 Bar and 303 K with restraints to only the C-alpha atoms of the protein. Triplicate unrestrained MD simulations were run for 500 ns each. All water molecules were kept rigid using SETTLE[9]. Trajectories were analysed using CPPTRAJ[10] and visualised using VMD[11].

1 Schott-Verdugo, S. & Gohlke, H. PACKMOL-Memgen: A Simple-To-Use, Generalized Workflow for Membrane-Protein-Lipid-Bilayer System Building. *J Chem Inf Model* **59**, 2522-2528, (2019).

2 Yang, L. *et al.* Data for molecular dynamics simulations of B-type cytochrome c oxidase with the Amber force field. *Data Brief* **8**, 1209-1214, (2016).

3 Maier, J. A. *et al.* ff14SB: Improving the Accuracy of Protein Side Chain and Backbone Parameters from ff99SB. *J Chem Theory Comput* **11**, 3696-3713, (2015).

4 MacKerell, A. D. *et al.* All-atom empirical potential for molecular modeling and dynamics studies of proteins. *J Phys Chem B* **102**, 3586-3616, (1998).

5 Case, D. A. *et al.* The Amber biomolecular simulation programs. *J Comput Chem* **26**, 1668-1688, (2005).

6 Salomon-Ferrer, R., Case, D. A. & Walker, R. C. An overview of the Amber biomolecular simulation package. *Wires Comput Mol Sci* **3**, 198-210, (2013).

7 Salomon-Ferrer, R., Gotz, A. W., Poole, D., Le Grand, S. & Walker, R. C. Routine Microsecond Molecular Dynamics Simulations with AMBER on GPUs. 2. Explicit Solvent Particle Mesh Ewald. *J Chem Theory Comput* **9**, 3878-3888, (2013).

8 Berendsen, H. J. C., Postma, J. P. M., Vangunsteren, W. F., Dinola, A. & Haak, J. R. Molecular-Dynamics with Coupling to an External Bath. *J Chem Phys* **81**, 3684-3690, (1984).

9 Miyamoto, S. & Kollman, P. A. Settle - an Analytical Version of the Shake and Rattle Algorithm for Rigid Water Models. *Journal of Computational Chemistry* **13**, 952-962, (1992).

10 Roe, D. R. & Cheatham, T. E. PTRAJ and CPPTRAJ: Software for Processing and Analysis of Molecular Dynamics Trajectory Data. *Journal of Chemical Theory and Computation* **9**, 3084-3095, (2013).

11 Humphrey, W., Dalke, A. & Schulten, K. VMD: Visual molecular dynamics. *J Mol Graph Model* **14**, 33-38, (1996).

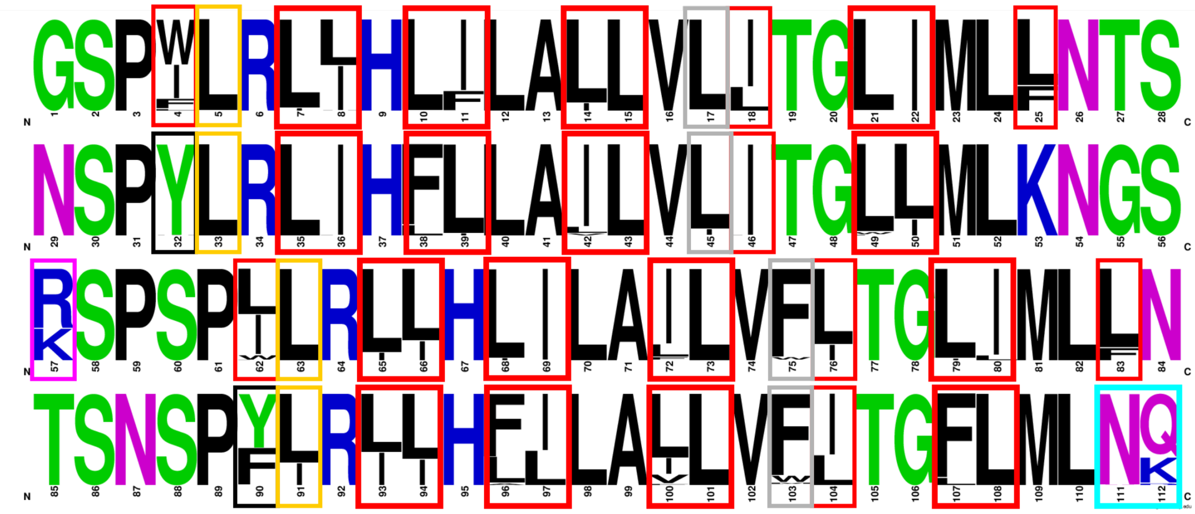

Figure S1: Sequence logo presentation of the multiple sequence alignment of the 200 best-scoring decoys from Rosetta design. Key: *Red box*, position allowed to sample sequence alphabet FAILVWGST. *Grey box,* Y in original 4D2 sequence restricted to non-polar FWILVM. *Black box*, E in original 4D2 sequence restricted to aromatic sidechains YFW. *Yellow box*, restricted alphabet ILV to ensure hydrophobic helix ends. *Magenta box*, allow positive residues KR in loop 2 to promote N-in/C-in topology. *Cyan box*, allow positive and polar residues KRNQ to promote N-in/C-in topology. Image created with Weblogo[1] (https://weblogo.threeplusone.com)

1 Crooks, G. E., Hon, G., Chandonia, J. M. & Brenner, S. E. WebLogo: a sequence logo generator. *Genome Res* **14**, 1188-1190, (2004).

```
50_289      MGSPILRIIHLILALLVLITGLIMLLNTSNSPYLRLIHFLLALLVLITGWLMLKNGSKSP    60
49_13       MGSPFLRLIHLILALLVLLTGLIMLLNTSNSPYLRLIHFLLAILVLITGLIMLKNGSRSP    60
30_82       MGSPWLRLLHLFLALLVLLTGLIMLLNTSNSPYLRLIHFLLAILVLITGLLMLKNGSRSP    60
            **** **::**:******:***********************:****** :******:**

50_289      SPILRLIHIILAILVFITGIIMLLNTSNSPFLRILHFILALLVFITGFLMLNQ   113
49_13       SPILRLLHLILAILVFLTGLIMLWNTSNSPYLRLIHFLLALLVFLTGFLMLNQ   113
30_82       SPLLRLLHLILAILVFLTGLIMLLNTSNSPYLRLLHLILALLVFITGFLMLNQ   113
            **:***:*:*******:**:*** ******:**::*::******:********
```

Figure S2: **Comparison of surface-swapped amino acid sequences produced by Rosetta design.** Sequences are identified by their arbitrary Rosetta simulation ID and aligned in ClustalOmega. 50-289 is renamed CytbX in the accompanying paper.

Figure S3: Synthetic genes corresponding to each protein design. The translation of each gene is shown directly underneath.

>CytbX_synthetic_gene
ATGGGCTCTCCTATTCTGCGCATCATTCACCTGATTTTGGCCTTGCTGGTTCTGATTACCGGACTTATCATGCTG
CTGAATACGTCAAATAGCCCCTATCTTCGCCTCATTCATTTTTTACTGGCACTGCTCGTGCTGATTACCGGTTGG
CTGATGCTAAAAAACGGTAGTAAGAGTCCGAGCCCGATCCTCCGTTTAATCCACATAATTCTGGCAATACTGG
TATTTATTACTGGCATCATTATGTTACTGAACACATCGAACAGCCCATTCCTGCGGATTTTGCATTTCATCCTTG
CGTTATTGGTCTTTATCACGGGCTTCCTTATGCTGAACCAGGCGGCCGCAGGTAAACCGATCCCGAATCCACT
GTTAGGGCTGGATTCCACCCATCACCACCATCACCATCACCATCATCAT**TGA**

>CytbX_translated
MGSPILRIIHLILALLVLITGLIMLLNTSNSPYLRLIHFLLALLVLITGWLMLKNGSKSPSPILRLIHIILAILVFITGIIMLLNT
SNSPFLRILHFILALLVFITGFLMLNQAAAGKPIPNPLLGLDSTHHHHHHHHHH*

>CytbX-GFP_gene
ATGGGCTCTCCTATTCTGCGCATCATTCACCTGATTTTGGCCTTGCTGGTTCTGATTACCGGACTTATCATGCTG
CTGAATACGTCAAATAGCCCCTATCTTCGCCTCATTCATTTTTTACTGGCACTGCTCGTGCTGATTACCGGTTGG
CTGATGCTAAAAAACGGTAGTAAGAGTCCGAGCCCGATCCTCCGTTTAATCCACATAATTCTGGCAATACTGG
TATTTATTACTGGCATCATTATGTTACTGAACACATCGAACAGCCCATTCCTGCGGATTTTGCATTTCATCCTTG
CGTTATTGGTCTTTATCACGGGCTTCCTTATGCTGAACCAGGCGGCCGCAGGTAAACCGATCCCGAATCCACT
GTTAGGGCTGGATTCCACCCTCGAGCTGGTGCCGCGCGGCAGCAGTAAAGGAGAAGAACTTTTCACTGGAGT
TGTCCCAATTCTTGTTGAATTAGATGGTGATGTTAATGGGCACAAATTTTCTGTCCGTGGAGAGGGTGAAGGT
GATGCTACAAACGGAAAACTCACCCTTAAATTTATTTGCACTACTGGAAAACTACCTGTTCCGTGGCCAACACT
TGTCACTACTCTGACCTATGGTGTTCAATGCTTTTCCCGTTATCCGGATCACATGAAACGGCATGACTTTTTCAA
GAGTGCCATGCCCGAAGGTTATGTACAGGAACGCACTATATCTTTCAAAGATGACGGGACCTACAAGACGCG
TGCTGAAGTCAAGTTTGAAGGTGATACCCTTGTTAATCGTATCGAGTTAAAGGGTATTGATTTTAAAGAAGAT
GGAAACATTCTTGGACACAAACTGGAGTACAACTTTAACTCACACAATGTATACATCACGGCAGACAAACAAA
AGAATGGAATCAAAGCTAACTTCAAAATTCGCCACAACGTTGAAGATGGTTCCGTTCAACTAGCAGACCATTA
TCAACAAAATACTCCAATTGGCGATGGCCCTGTCCTTTTACCAGACAACCATTACCTGTCGACACAATCTGTCC
TTTCGAAAGATCCCAACGAAAAGCGTGACCACATGGTCCTTCTTGAGTTTGTAACTGCTGCTGGGATTACACAT
GGCATGGATGAGCTCTACAAACTCGAACACCACCACCACCACCACCACCACCAC**TGA**

>CytbX-GFP_gene_translated
MGSPILRIIHLILALLVLITGLIMLLNTSNSPYLRLIHFLLALLVLITGWLMLKNGSKSPSPILRLIHIILAILVFITGIIMLLNT
SNSPFLRILHFILALLVFITGFLMLNQAAAGKPIPNPLLGLDSTLELVPRGSSKGEELFTGVVPILVELDGDVNGHKFSV
RGEGEGDATNGKLTLKFICTTGKLPVPWPTLVTTLTYGVQCFSRYPDHMKRHDFFKSAMPEGYVQERTISFKDDGT
YKTRAEVKFEGDTLVNRIELKGIDFKEDGNILGHKLEYNFNSHNVYITADKQKNGIKANFKIRHNVEDGSVQLADHY
QQNTPIGDGPVLLPDNHYLSTQSVLSKDPNEKRDHMVLLEFVTAAGITHGMDELYKLEHHHHHHHHHH-

>49_13_synthetic_gene
ATGGGGTCGCCTTTCCTGCGCCTGATTCATCTGATCCTCGCTCTGTTGGTGCTGCTTACCGGCCTGATTATGTT
ACTTAATACGAGCAACAGTCCATATCTCCGGTTGATCCACTTCCTACTGGCCATTCTAGTCCTCATTACCGGCCT
GATAATGCTGAAAAATGGTTCACGTAGTCCGAGCCCAATTCTGCGTCTTTTGCACCTGATCTTAGCAATTCTGG
TATTTCTGACGGGTTTGATCATGTTATGGAACACTTCCAACTCTCCGTACTTGCGCCTGATTCATTTTCTTTTAG
CGCTGTTAGTTTTTCTGACAGGCTTCCTGATGCTGAACCAGGCGGCCGCAGGTAAACCGATCCCGAATCCCCT
GCTCGGACTGGATAGCACCCATCATCATCACCATCACCATCATCACCAC**TGA**

>49_13_translated
MGSPFLRLIHLILALLVLLTGLIMLLNTSNSPYLRLIHFLLAILVLITGLIMLKNGSRSPSPILRLLHLILAILVFLTGLIMLW
NTSNSPYLRLIHFLLALLVFLTGFLMLNQAAAGKPIPNPLLGLDSTHHHHHHHHHH*

>30_82_synthetic_gene
ATGGGCTCTCCGTGGTTACGGCTTCTGCACCTGTTTTTAGCATTATTGGTACTGCTCACCGGCCTCATCATGCTT
CTGAACACTAGTAATTCGCCCTACCTGCGCCTGATACACTTCTTGTTAGCTATTCTGGTTTTAATTACGGGTCTG
CTGATGCTGAAAAACGGCAGCCGTTCACCGAGCCCGCTCCTACGTCTACTGCACTTGATTCTGGCGATCCTGG
TGTTTTTAACGGGGCTTATTATGCTGCTGAACACATCCAATAGTCCATATCTTCGCCTTCTGCATCTGATCCTGG
CCCTGCTGGTCTTTATCACCGGTTTCCTGATGCTCAATCAGGCGGCCGCAGGTAAACCGATTCCTAACCCATTG
TTGGGATTGGATAGCACCCACCATCACCACCATCATCATCATCATCAT**TGA**

>30_82_translated
MGSPWLRLLHLFLALLVLLTGLIMLLNTSNSPYLRLIHFLLAILVLITGLLMLKNGSRSPSPLLRLLHLILAILVFLTGLIML
LNTSNSPYLRLLHLILALLVFITGFLMLNQAAAGKPIPNPLLGLDSTHHHHHHHHHH*

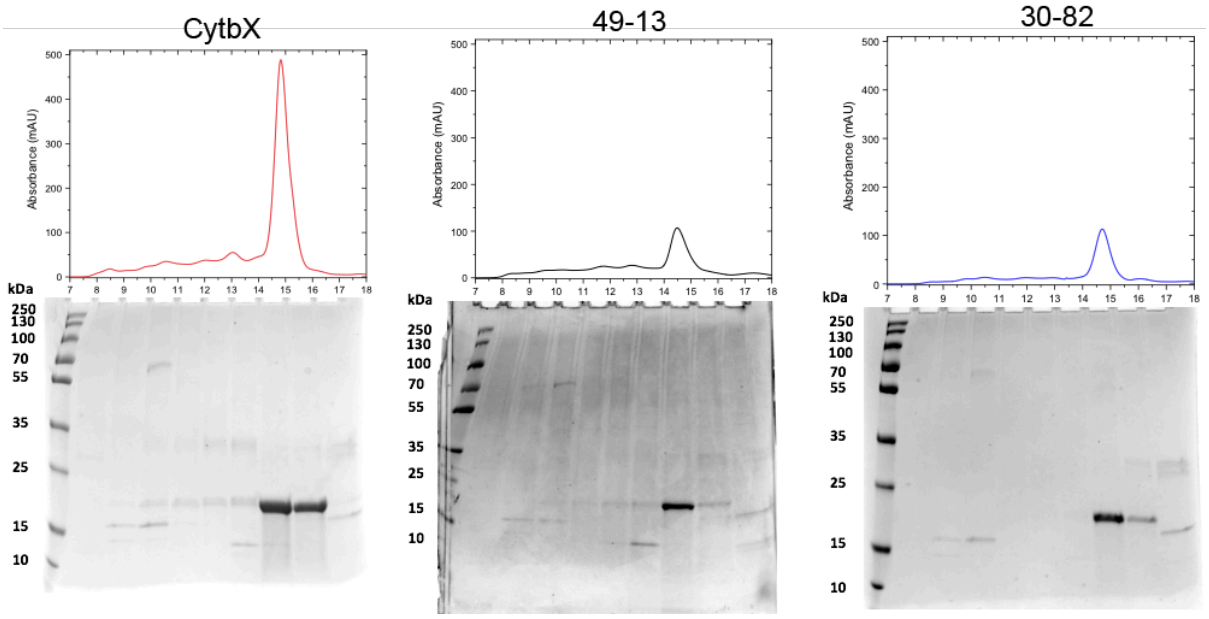

Figure S4. Purification of three Rosetta designs. *Top*, size-exclusion chromatography of designs solubilised from *E. coli* membranes in Cymal-5; *Bottom*, Coomassie-stained SDS-PAGE of column fractions. Data for CytbX are reproduced from main text. Two other designs, known only by their Rosetta IDs 49-13 and 30-82, are the cyan square and orange circle, respectively, on Fig. 1a. Both variants were successfully overexpressed and are monodisperse in Cymal-5 but give purification yields ~20% that of CytbX.

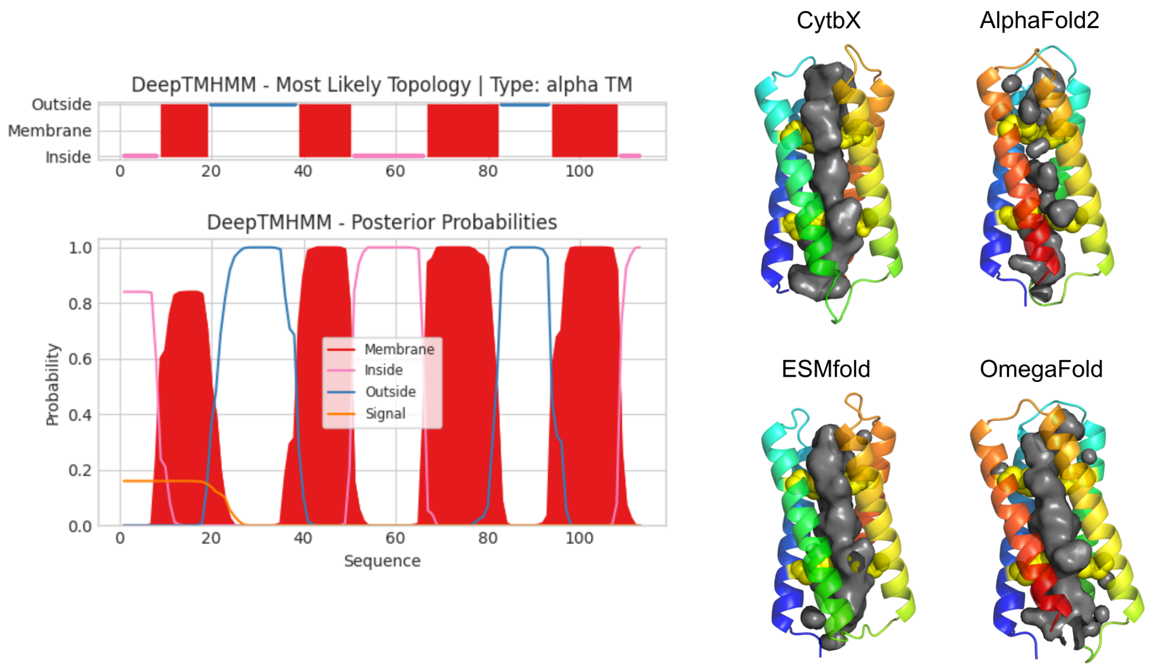

Figure S5. Topology prediction and *ab initio* structure prediction of CytbX. *Left panel* shows the results of a DeepTMHMM prediction of transmembrane topology, strongly suggesting that the CytbX sequence will form four transmembrane alpha helices in an N_{in}/C_{in} orientation. *Right panels* show CytbX structures as predicted by three different machine learning methods shown. AlphaFold2[1] predicts an alternative conformation but does not predict an internal cavity for heme binding; this same outcome was also observed for the original 4D2 construct and is at odds with the known experimental structure (PDB 7AH0). In contrast the language-based models ESMfold[2] and OmegaFold[3] do predict a heme cavity. ESMfold model differs from the CytbX design by only 0.6 Å.

1 Jumper, J. *et al.* Highly accurate protein structure prediction with AlphaFold. *Nature* **596**, 583-589, (2021).
2 Lin, Z. *et al.* Language models of protein sequences at the scale of evolution enable accurate structure prediction. *bioRxiv*, (2022).
3 Wu, R. *et al.* High resolution *de novo* structure prediction from primary sequence. *bioRxiv*, (2022).

Supplementary Figure 5.

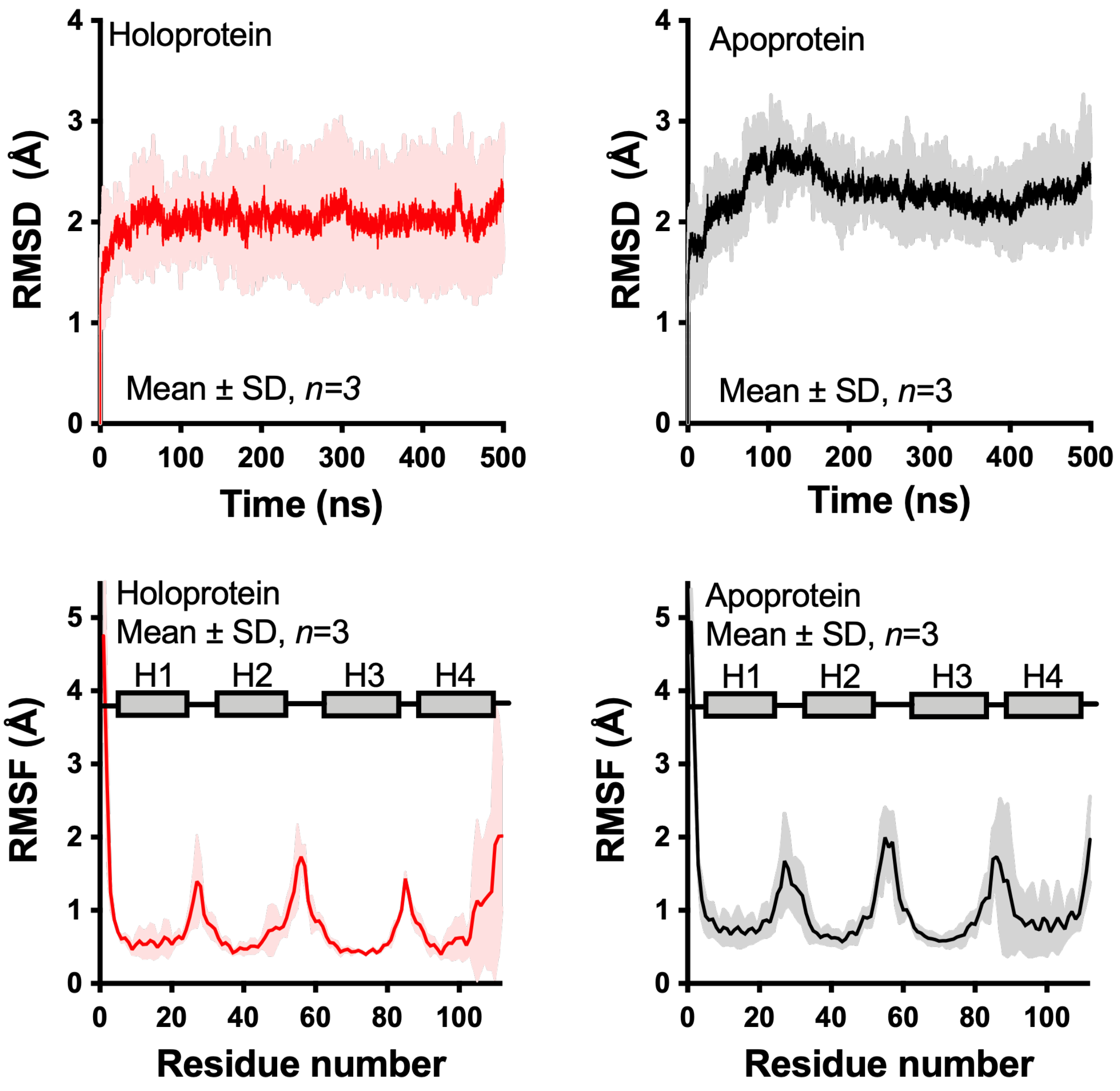

Figure S6. Molecular dynamics simulations of CytbX in an explicit bilayer. Cα RMSD plots show that both the apo- and holoprotein equilibrate rapidly and are stable thereafter. Backbone fluctuations are low in the helical regions of the protein, schematised as grey rectangles *H1-4* in the bottom panels. The apoprotein exhibits slightly greater fluctuation overall.
